# Biochemical Characterization of Cellulase From *Bacillus subtilis* Strain and its Effect on Digestibility and Structural Modifications of Lignocellulose Rich Biomass

**DOI:** 10.3389/fbioe.2021.800265

**Published:** 2021-12-20

**Authors:** Waseem Ayoub Malik, Saleem Javed

**Affiliations:** Department of Biochemistry, Faculty of Life Sciences, Aligarh Muslim University, Aligarh, India

**Keywords:** cellulolytic microbes, saccharification, biofuels, peptide sequencing, carboxymethyl cellulose

## Abstract

Microbial cellulases have become the mainstream biocatalysts due to their complex nature and widespread industrial applications. The present study reports the partial purification and characterization of cellulase from *Bacillus subtilis* CD001 and its application in biomass saccharification. Out of four different substrates, carboxymethyl cellulose, when amended as fermentation substrate, induced the highest cellulase production from *B. subtilis* CD001. The optimum activity of CMCase, FPase, and amylase was 2.4 U/ml, 1.5 U/ml, and 1.45 U/ml, respectively. The enzyme was partially purified by (NH_4_)_2_SO_4_ precipitation and sequenced through LC-MS/MS. The cellulase was found to be approximately 55 kDa by SDS-PAGE and capable of hydrolyzing cellulose, as confirmed by zymogram analysis. The enzyme was assigned an accession number AOR98335.1 and displayed 46% sequence homology with 14 peptide-spectrum matches having 12 unique peptide sequences. Characterization of the enzyme revealed it to be an acidothermophilic cellulase, having an optimum activity at pH 5 and a temperature of 60°C. Kinetic analysis of partially purified enzyme showed the Km and Vmax values of 0.996 mM and 1.647 U/ml, respectively. The enzyme activity was accelerated by ZnSO_4,_ MnSO_4,_ and MgSO_4,_ whereas inhibited significantly by EDTA and moderately by β-mercaptoethanol and urea. Further, characterization of the enzyme saccharified sugarcane bagasse, wheat straw, and filter paper by SEM, ATR-FTIR, and XRD revealed efficient hydrolysis and structural modifications of cellulosic materials, indicating the potential industrial application of the *B. subtilis* CD001 cellulase. The findings demonstrated the potential suitability of cellulase from *B. subtilis* CD001 for use in current mainstream biomass conversion into fuels and other industrial processes.

## Highlights

• The cellulase from *B. subtilis* CD001 displayed 46% sequence similarity with the master protein with a molecular weight of 55 kDa.
• The enzyme was observed to be thermophilic cellulase with desirable temperature and pH stability for industrial use.
• 30–40% biomass saccharification was achieved under indigenous cellulase treatment.
• SEM, FTIR, and XRD investigations revealed the high efficacy of the enzyme towards biomass digestibility.

## Introduction

Lignocellulose-rich biomass and agricultural waste are abundant and widespread sources of carbon in nature. Production of biofuel and value-added products from lignocellulosic biomass (LCB) is gaining impetus worldwide (Philippini et al., 2020). However, the impediment in exploiting the LCB for biofuel production lies in the crystalline nature of cellulose enclosed by hemicellulose and lignin components. The complex structure formed by these components limits the efficient hydrolysis and therefore leads to low degradation in digestion processes (Liang et al., 2018). The deconstruction of the recalcitrant structure of biomass materials could be achieved by the process of pretreatment to improve the accessibility to cellulose. A common classification separates different pretreatments into physical, chemical, physicochemical, and biological treatments (Ravindran and Jaiswal, 2016). Biological pretreatment to LCB employing microorganisms or their enzymes offers an eco-friendly process for biomass processing. This method is achieving much importance as it can be performed under mild conditions, consume less energy, and hardly generates any toxic substances, thus offering an environmentally sound process (Baramee et al., 2020; Zhang et al., 2020). Cellulolytic and xylanolytic enzymes greatly contribute in the conversion of agro-based LCB into valuable products i.e. biofuels (Ezeilo et al., 2020). For the proficient hydrolysis of biomass materials, the combined stroke of both enzymes is indispensable (Philippini et al., 2020). Cellulases and hemicellulases find their application in hydrolyzing the LCB with the aim of producing monomeric sugars that are easily utilizable for fermentation by microorganisms (Jin et al., 2020). Both bacteria and fungi are capable of secreting cellulolytic enzymes. Mostly the bacterial species have been used predominantly in industrial processes, wherein the *Bacillus* genus occupied a prime space for secreting a collection of hydrolytic enzymes (Kumar and Singh, 2021). Bacteria are prolific producers of cellulases as these enzymes are extracellularly secreted in abundant amounts, facilitating easy extraction and purification processes. The cellulolytic enzymes from *B. subtilis* have been studied thoroughly for their enough production and application for biomass valorization (Deka et al., 2011). Bacterial species, such as *B. subtilis subsp. subtilis* JBS250 (Kumar and Singh, 2021), *B. subtilis* strain LFS3 (Rawat and Tewari, 2012), *B. subtilis* Strain CBS31 (Sudip et al., 2020), *Bacillus halodurans* IND18 (Vijayaraghavan et al., 2016), *B. subtilis* BC1 (Dehghanikhah et al., 2020), *B. subtilis* BY-4 (Ma et al., 2015), and *B. subtilis* CD001 (Malik et al., 2021) have been recently reported for cellulase production. Bacteria reveal a wide variety of physicochemical conditions. The bacteria screened from the environment harbor many properties like being thermophilic or psychrophilic, alkaliphilic, or acidophilic. This spacious diversity obtained by the microbes helps out in screening the efficient cellulase producing bacteria to overcome the present challenges associated with industrial processes (Rastogi et al., 2010; Irfan et al., 2017; Dehghanikhah et al., 2020; Kumar and Singh, 2021). Enzymes with high-temperature stability over extended periods have proved to be very efficient in industrial biomass processes and are better suited to potential enzyme recovery and recycling (Jin et al., 2020). The relevance gained by the cellulolytic microbes and their enzymes in the industrial field with the purpose of generating bioethanol from LCB creates a center of attention (Tsegaye et al., 2018). The lignocellulosic substrates like sugarcane bagasse, wheat straw, paddy straw, banana fruit stalk, and other biomass residues are used as potential stocks for cellulase production and their subsequent action for hydrolyzing these biomass materials (Pedersen and Meyer, 2009; Vijayaraghavan et al., 2016; Matei et al., 2020). Advances in biological pretreatment methods applied to LCB and some other wastes are still important to understand the mechanisms of the process. This will improve economic viability within available biomass and enzymes and keep the process as eco-friendly as possible (Matei et al., 2020).

As stated above, this study describes partial purification and characterization of cellulase from *B. subtilis* CD001 and its potential ability to disintegrate the lignocellulosic biomass residues. The temperature and pH stability of the enzyme have been studied comprehensively, along with its tolerance to different additives. Furthermore also proposed here is a robust indigenous cellulolytic pretreatment strategy to enhance the degradation of sugarcane bagasse, wheat straw, and filter paper. The alterations in lignocellulosic materials were examined in terms of chemical composition, morphology, and physical structure. The efficacy of the treatment process was measured in terms of glucose formation from cellulose. In addition to being environment friendly, the biological treatment strategy could be of commercial interest as well.

## Materials and Methods

### Microorganism, Reagents, and Preparations of Lignocellulosic Materials

“*Bacillus subtilis*”, A potential cellulase producing bacteria was successfully isolated from cow dung as described earlier (Malik et al., 2021). The nucleotide sequence was deposited to NCBI GeneBank and an accession number MN173007 was obtained. It is also preserved at CSIR-Institute of Microbial Technology, Chandigarh (India), under deposit number MTCC12940 (Malik et al., 2021). The bacteria was routinely cultured on CMC agar medium, sub-culturized at regular intervals, and kept at 4°C during their storage time. Carboxymethyl cellulose (CMC), MgSO_4_, (NH_4_)_2_SO_4_, KH_2_PO_4_, yeast extract, galactose, were procured from Sisco Research Laboratories (Mumbai, India). Whatman Filter Paper Grade 1 (FP) and all other chemicals used for the protein purification process were of laboratory grade and purchased from Merck (Mumbai, Maharashtra, India). Sugarcane bagasse (SCB) and wheat straw (WS) were collected from the neighboring area of AMU, Aligarh U.P., India. The lignocellulosic materials (SCB, WS, and FP) 10 g of each were cut into small pieces approximately of the same size (2 × 5 mm). The shredded SCB, FP, and WS were soaked in autoclaved distilled water and kept on a magnetic stirrer to remove all the impurities and also the glucose content if present. A further step was to air-dry these ground-washed substrates completely until they contain no more than 10% of moisture content. After that, all the three substrates were sterilized at 121°C for 30 min and stored at room temperature before being used for biological pretreatment (Singh et al., 2008).

### Quantification of Cellulase Production on Different Carbon Sources

The substrates, CMC, SCB, WS, and FP were analyzed to know their effect on cellulase production by the bacterium *B. subtilis* CD001 during the solid-state fermentation process (Sajith et al., 2014). The bacteria were allowed to grow in five separate conical flasks composed of 3.0 g/L galactose, 0.5 g/L sodium nitrate, 0.75 g/L KH_2_PO_4_, and 0.375 g/L MgSO_4_·7H_2_O. Each flask was supplemented with different carbon sources (1% w/v) as SCB, WS, FP, and CMC. One flask with no substrate was used as a control. For the sterilization process, all the flasks were autoclaved at 121°C for 15 min. Flasks were cooled to room temperature and were inoculated with 50 μl actively growing bacterial suspension. Incubation was carried out at 45°C on a shaker incubator at 120 rpm for 72 h. After 72 h of incubation, the supernatant was collected as per the previous protocol and analyzed for cellulase production in terms of CMCase activity (Malik et al., 2021). The culture medium together with carbon substrate that yielded maximum cellulase production was selected and used in further experiments.

### Cellulase Activity Profile of *B. subtilis* CD001

The cellulase activity profile of the enzyme was determined by using the crude enzyme source obtained as per the previous protocol (Malik et al., 2021). The cellulase activity was measured by the amount of reducing sugars released per mL of sample per min under assay conditions. Endoglucanase and exoglucanase activities were determined by the 3,5-Dinitrosalicylic acid method (DNS) (Miller, 1959) using CMC and FP as substrate, respectively (Zhang et al., 2020). The glucoamylase activity was estimated by the method followed by Sadalage et al. (2020) where 1% (w/v) starch was used as substrate. For all these enzyme assays, the reaction was carried out in 50 mM sodium citrate buffer (pH 4.8). Briefly, 0.5 ml of crude enzyme solution was added to 1% of each CMC, FP, and starch present in the buffer solution. The reaction was performed at 50°C for 30 min and stopped by adding 3 ml of DNS reagent. Soon after the addition of DNS all the samples were boiled for 5 min, cooled in water for color stabilization, and the optical density was measured at 540 nm. The absorbance was recorded against the blank at 540 nm using a UV-vis spectrophotometer (Shimadzu UV-1601; Japan). For regression analysis, the reactions were repeated three or more independent times, and the enzyme activities were reported and expressed as international units (IU). Specifically, 1 U of activity represents the amount of enzyme required to liberate 1 μmol of glucose equivalents under standard conditions (Bischoff et al., 2009).

### Partial Purification of the Enzyme

Of the four-carbon sources tested, the most promising carbon source was selected based on its role in enzyme production. *B. subtilis* was allowed to grow in the production medium optimized with the carbon source described above. The culture broth obtained from the 72 h incubation was centrifuged at 5,000 rpm at 4°C for 15 min to separate the bacterial biomass from the supernatant. The supernatant was then saturated with (NH_4_)_2_SO_4_ at 80% saturation overnight at 4°C to precipitate the proteins. The sample was centrifuged at 12,000 rpm for 30 min at 4°C to collect the pellet as a protein sample. The pellet obtained was resuspended in 50 mM sodium citrate buffer (pH 4.8) and dialyzed overnight against the same buffer at 4°C (Saini et al., 2020). The dialyzed sample was rechecked for cellulase activity profile, and the protein content was estimated by the Lowry method (Lowry, 1990). The molecular weight of the partially purified cellulase was determined by 12% sodium dodecyl sulfate-polyacrylamide gel electrophoresis (SDS-PAGE), and the cellulase conformity was done by zymogram analysis (Yu and Li, 2015). The molecular ladder (5–205 kDa) (Thermo Fisher Scientific) was used to determine the molecular weight of the partially purified enzyme. The activity of the enzyme protein was confirmed by zymogram analysis on 10% native-PAGE using CMC (1%) as substrate. After the run at 70 V for 1.5 h, the gel with protein marker was stained with Coomassie Brilliant Blue dye (CBB-R 250) and de-stained with glacial acetic acid: methanol: water (10:10:80). For zymogram analysis, the native gel was properly washed with sodium citrate buffer and then incubated in a water bath at 50°C for 10 min. This was followed by transfer of gel to 0.1% Congo red solution for 10 min and then de-staining with 1% NaCl until the CMCase activity was visualized as a clear band against the red background (Sajith et al., 2014; Sadalage et al., 2020).

### Mass Spectrometric Analysis of Peptide Mixtures

#### Sample Preparation

Prior to peptide analysis, a single subunit gel band obtained was subjected to in-gel digestion procedures during which gel band was cut and reduced with 5 mM TCEP (tris (2-carboxyethyl) phosphine). Before trypsin (1:50, Trypsin/lysate ratio) digestion for 16 h at 37°C, the gel band was further alkylated with 50 mM iodoacetamide (Petkauskaite et al., 2014). Digested materials were cleaned with a C18 silica cartridge to remove the salt. The speedVac dried pellet was then resuspended in buffer (5% acetonitrile, 0.1% formic acid). This experiment was performed using EASY-nLC 1200 system (Thermo Fisher Scientific) coupled to Thermo Fisher-QExactive equipped with a nano-electrospray ion source. Briefly, 1.0 µg of the peptide mixture was resolved using 25 cm PicoFrit column (360 µm outer diameter, 75 µm inner diameter, 10 µm tip) filled with 1.9 µm of C18-resin (Dr. Maeisch, Germany). The peptides were loaded with buffer (5% acetonitrile, 0.1% formic acid) and eluted with a 0–40% gradient of buffer (95% acetonitrile, 0.1% formic acid) at a flow rate of 300 nl/min for 60 min. The MS data were obtained using a data-dependent top10 method dynamically choosing the most abundant precursor ions from the survey scan (Panda et al., 2020).

### Data Processing

The raw data generated from all samples were analyzed by Proteome Discoverer (v2.2) against the *B. subtilis* (strain 168) protein sequence database. The precursor and fragment mass tolerances for Sequence and Amanda were set to 10 ppm and 0.5 Da, respectively. In order to generate peptides, a protease specificity of trypsin/P was selected (cleavage at the C terminus of K/R, unless followed by “P”) and with a maximum missed cleavage value of two. For database searching, carbamidomethyl on cysteine was considered a fixed modification, and methionine oxidation and N-terminal acetylation were considered a variable modification. Protein false discovery rate and peptide spectrum match were both set to 0.01 False Discovery Rate (FDR).

### Effect of pH and Temperature on the Activity of Partially Purified Cellulase

Temperature and pH play a significant role in determining the specificity and efficiency of the enzyme for substrate hydrolysis (Rawat and Tewari, 2012; Dehghanikhah et al., 2020). In this study, the effect of pH on the partially purified cellulase was assessed by measuring the activity in 50 mM sodium citrate buffer between the pH 3.0–9.0, using 1% CMC as substrate. The effect of temperature on the enzyme activity was also ascertained by incubating the reaction at temperatures varying from 30°C to 70°C in the same buffer of pH 4.8. In both cases, the reaction mixture contains 0.5 ml of enzyme added to 0.5 ml of 1% CMC and the reaction was carried out for 30 min of incubation time. The reaction was stopped by adding 3 ml of DNS, boiled for 5 min, and thereafter cooled at room temperature. Cellulase activity was determined as discussed above. The pH stability of the cellulase was determined by pre-incubating the enzyme in sodium citrate buffer from pH 3.0 to 9.0 for 24 h at room temperature. While as for thermal stability, the enzyme alone dissolved in 50 mM sodium citrate buffer (pH 4.8) was also pre-incubated at different temperatures ranging from 30 to 70°C for 72 h before the cellulase activity profile was monitored (Jin et al., 2020).

### Determination of Km and Vmax

The Michaelis-Menten kinetic constants, Km and Vmax for partially purified cellulase, were determined by measuring the enzyme activity at different concentrations of CMC ranging from 0.25 to 3.0%. These reactions were also performed in 50 mM sodium citrate buffer of pH 5.0. Lineweaver-Burk plot was used to determine the exact values of Km and Vmax (Walia et al., 2014). Briefly 0.5 ml of varying concentrations of CMC (0.25–3.0%) in 0.5 M citrate buffer (pH 5.0) was added to 0.5 ml of partially purified cellulase. The reaction samples were incubated at 60°C for 30 min, and the activity was determined by the DNS method.

### Effect of Different Additives on Enzyme Activity

Various additives like metal ions, inhibitors, and surfactants were employed to evaluate their effect on the CMCase activity at 60°C and pH of 5.0. The metal ions employed in this study were NaCl, KCl, ZnSO_4_, MgSO_4_, MnSO_4_, HgCl_2_, CaCl_2_, FeSO_4_, AlSO_4_, and FeCl_3_ with a concentration of 10 mM (Jin et al., 2020). The inhibitors, β-mercaptoethanol (βME), Ethylenediaminetetraacetic acid (EDTA) as well as urea were evaluated at a concentration of 5 mM. Surfactants like SDS and Triton X-100 were employed at a concentration of 1%. All the additives mentioned were separately mixed with the enzyme in 50 mM sodium citrate buffer at pH 5.0. The reaction mixtures were separately incubated at 37°C for 30 min (Kumar and Singh, 2021). Then after the individual reactions were carried out with 1% of CMC, and relative activity was estimated by the DNS method. In all the cases, the activity measures without the addition of any compound were taken as control (100%) (Walia et al., 2014).

### Saccharification of Cellulase Treated Biomass Residues

The hydrolytic activities of the crude enzyme with different substrates were analyzed by measuring the percentage of glucose liberated from different substrates used. For saccharification, all the prepared substrates, CMC, SCB, FP, and WS without any pretreatment process, were directly subjected to crude enzymatic treatment (Singh et al., 2008; Tabssum et al., 2018; Philippini et al., 2020). Saccharification assays were performed in triplicates, in 50 ml Erlenmeyer flasks sealed with latex caps, using a substrate load of 5.0% (dry weight basis). Enzyme load of 1 U/ml on each prepared substrate and sodium citrate solution (50 mM, pH 4.8) added with the ratio of 1:1. Flasks were maintained at 50°C and 100 rpm in a water bath for 72 h. After 72 h of incubation, the mixture was cooled to room temperature, and the contents were filtered through a muslin cloth. The liquid phase was filtered through 0.22-micron filters and then analyzed for glucose content using the DNS method. The concentration of sugar molecules liberated at different times was determined by using the glucose standard curve. Glucose yield (%) was recorded using CMC as reference (100%). Saccharification (%) was calculated using the following formulae (Irfan et al., 2017; Prajapati et al., 2020).

%Hydrolysis efficiency=Reducing Sugars released(mg/mL)×100/Substrate used(mg/mL).

After the enzymatic treatment, all the samples were air-dried and analyzed for morphological changes via Scanning electron microscopy (SEM), attenuated total reflection-Fourier transform infrared spectroscopy (ATR-FTIR), and X-ray diffraction (XRD) techniques.

### Structural and Crystallinity Measurement of Cellulase Treated Biomass Residues

The substrates that gave the promising percent saccharification results during enzymatic treatment were further chosen for detailed morphological characterizations. The morphology and the alteration in the surface functional groups of enzyme-treated substrates were studied using SEM and ATR-FTIR, respectively. Based on available evidence, these methods have been used to demonstrate the chemical and structural changes in biomass using diverse enzymatic saccharification (Liang et al., 2018; Saini et al., 2020). For SEM, the samples were air-dried, placed on carbon tape, and then sputter-coated with a gold film of 60 nm. The samples were examined under a scanning electron microscope (JOEL JSM-5310 SEM, Tokyo, Japan) at University Sophisticated Instruments Facility (USIF) AMU, Aligarh, India. FTIR analysis required preparing pellets containing moisture-free SCB, FP, and WS, with 3 mg potassium bromide. As the treated samples were oven-dried (45°C), FTIR spectroscopy was performed (PerkinElmer, American) using a spectrum range of 4,000 to 500 cm^−1^ with 40 scans per sample. The spectra were analyzed using the FTIR database. The data thus obtained was evaluated for chemical and structural changes that occurred in LCB using the reference peaks available in the literature (Liang et al., 2018; Baramee et al., 2020). In addition, the crystallinity index (CrI) was analyzed by XRD using an Ultima IV apparatus (Rigaku Corporation, Japan). The pretreated biomass was crushed and sieved at or below 200 mesh before it was analyzed. Samples spectra were recorded between 10° and 7° of 2θ (scattering angle) and at a scanning speed of 2°/ min. Calculation of the crystallinity index was done using the formula listed below (da Silva et al., 2018; Saini et al., 2020).

$$CrI=I_{002}-I_{am}\times 100/I_{002},$$

where I_00_2 denotes the maximum intensity of the diffraction at approximately 22.5° that relates to crystalline regions, and I_am_ designated the minimum intensity of the peak at approximately 18.0° that represents the amorphous regions.

### Statistical Analysis

The experiments were conducted in triplicate, and the results are expressed as the standard deviation of the mean (SDM) for three independent experiments. Statistically significant differences between groups were determined using one-way ANOVA, with a *p*-value less than 0.05 considered significant.

## Results and Discussion

### Optimization of Cellulase Production and Cellulase Activity Profile

**FIGURE 1:**
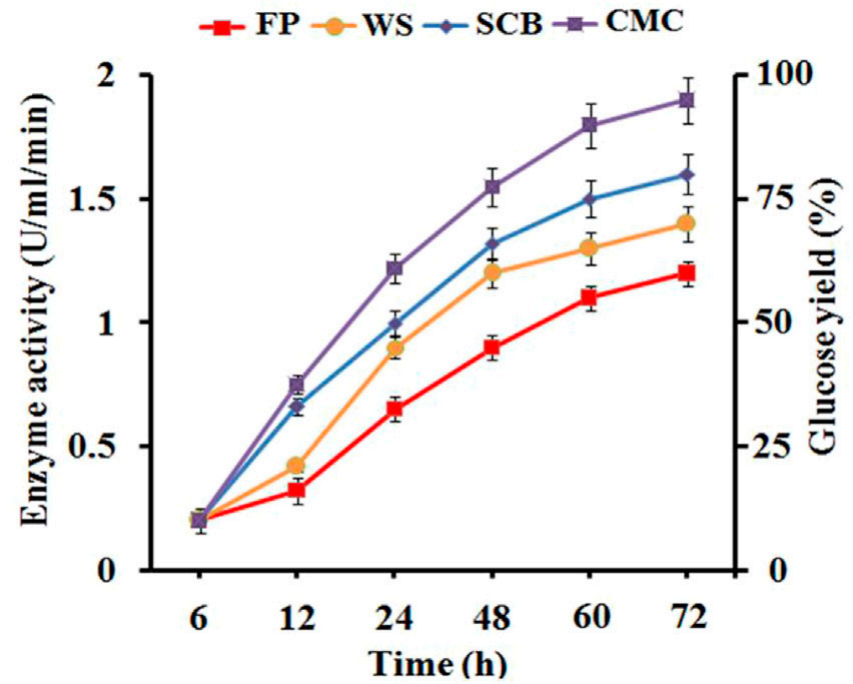
Cellulase production was measured in terms of CMCase activity in the culture supernatants during the addition of different carbon sources (CMC, SCB, WS, and FP) to the culture medium and the percentage of glucose released from these substrates upon crude cellulase treatment at distinct times.

The production profile of cellulolytic enzyme by *B. subtilis* CD001 was estimated in the optimized medium supplemented with 1% of different carbon sources. Among them, the highest cellulase production reported in terms of CMCase activity was reported for CMC. It was observed that all the four substrates viz, CMC, SCB, FP, and WS have the potential to stimulate the bacteria for cellulase production. Initially, linear enzyme production was observed from all the carbon sources. Still, at 72 h of incubation, CMC was found to be a major factor responsible for maximum cellulase production (Figure 1). Earlier also, CMC has been reported as the better carbon source inducing extracellular cellulase production with respect to other carbon sources (da Silva et al., 2021). The CMC induced cellulase production to 1.85 U/ml/min, however, cellulase production with SCB, WS, and FP was found to be lesser, i.e., 1.47 U/ml/min, 1.31 U/ml/min, and 0.89 U/ml/min, respectively. These results support the idea that natural carbon sources can also induce *B. subtilis* CD001 for cellulase production, though to a lesser extent. This is in accordance with the results of Sajith et al. (2014) who reported CMC as the best inducer for cellulase production with respect to other natural substrates used in their study. The crude enzyme obtained was analyzed for endoglucanase and exoglucanase activities using CMC and FP as substrates, respectively. A high titer of endoglucanase or CMCase (2.4 U/ml) was observed. The FPase or exoglucanase activity was noticed to be 1.5 U/ml.

In order to scrutinize the enzyme to attain the maximum degradation of the LCB, the crude enzyme was further analyzed for amylolytic activity. It was observed that the enzyme obtained from the strain *B. subtilis* CD001 harbor the property of glucoamylase (1.45 U/ml). In the present study, the isolate produces a relatively much higher titer of CMCase or endoglucanase or FPase, exoglucanase compared to other *Bacillus* strains reported (Rastogi et al., 2010). The potential of the enzyme having synergistic enzyme activities is helpful in achieving more efficacies in biomass saccharification (Agrawal et al., 2018). For industrial purposes, screening the bacterial enzyme with broader enzyme specificity is of much importance. The enzyme from *B. subtilis* CD001 also harbors spacious substrate specificity.

### SDS-PAGE and Zymogram Analysis

**FIGURE 2:**
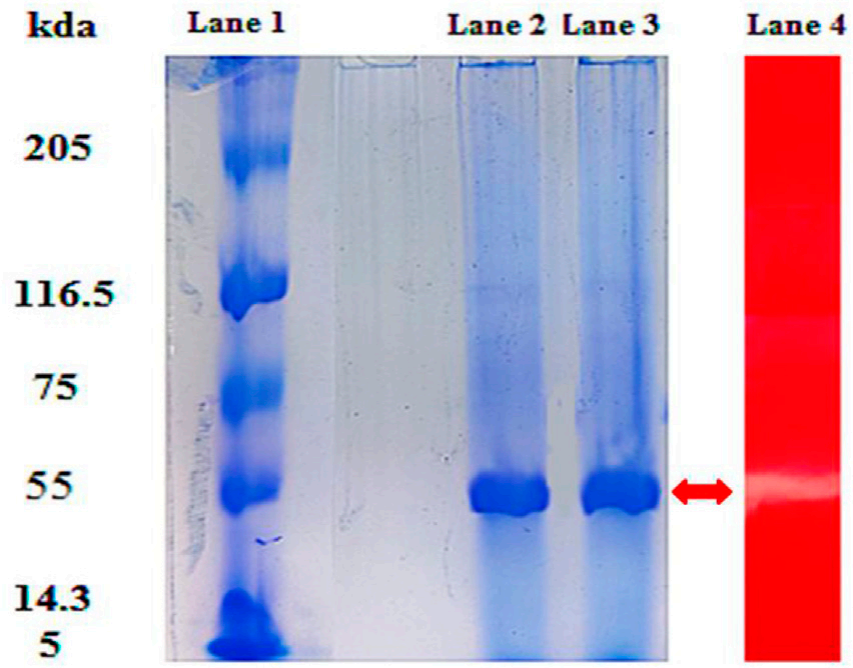
SDS-PAGE and zymogram analysis of cellulase. Lane 1 represents standard molecular weight marker proteins in kDa. Lane 2 and Lane 3 show a single band at approximately 55 kDa, and Lane 4 (L4) shows the zymogram of partially purified cellulase.

The cellulase enzyme was partially purified using the crude culture obtained from the optimized CMC broth medium inoculated with bacterial strain *B. subtilis* CD001. Crude enzyme obtained as a cell-free supernatant was precipitated using 80% (NH_4_)_2_SO_4_ saturation. The partially purified fraction was subjected to SDS and native PAGE analysis. The qualitative analysis of the enzyme performed through SDS-PAGE resulted in a single band with a molecular weight of about 55.0 kDa (Figure 2). The protein concentration at 80% saturation after dialysis was found to be 1.2 mg/ml. According to the SDS-PAGE profile of the partially purified cellulase, no other subunit was observed, and therefore, the enzyme was considered to be homogenous. These results are in agreement with the fact that most of the bacterial cellulases are found to be monomers, unlike fungal cellulases (Swathy et al., 2020). The results of the zymogram test showed a visible band of CMCase activity, which correlated well with the molecular weight values obtained on PAGE. The molecular weight obtained is in conformity with the purified cellulase (55 kDa) by Ma et al. (2015) from *B. subtilis* BY-4. A novel cellulolytic bacterium *Acinetobacter junii* GAC 16.2 also secreted the single subunit cellulase enzyme with a molecular weight of 55 kDa (Banerjee et al., 2020).

### MS Analysis of Peptide Mixtures

**FIGURE 3:**
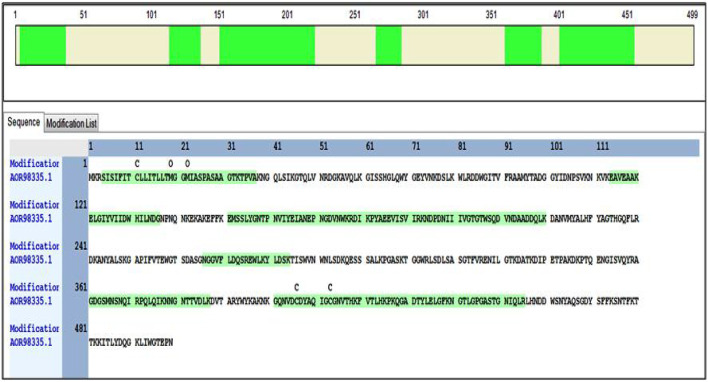
The green highlighted portion in the figure represents the amino acid sequence present in the protein of interest (cellulase) that hit the sequence of the master protein at different regions.

The molecular weight of the cellulase obtained from SDS-PAGE was approximately 55.2 kDa. Following the methods described, the band of interest was excised, treated, and digested. The purified enzyme cellulase was identified based on peptide fingerprinting extracted from *B. subtilis* CD001, using high throughput LC-MS/MS. The cellulase protein was assigned the accession NO. AOR98335.1 showed 46% sequence coverage with 14 peptide-spectrum matches and 12 unique peptides with the blast against the *B. subtilis* (strain 168) protein sequence database. Different peptides of the master protein (cellulase) were identified on modifications of carbamidomethyl, where 1× carbamidomethyl [C8]; 2× Oxidation [M16; M19] depicted the highest peptide-spectrum match (PSMs). The details of annotated sequences, Sequestand Amanda score, molecular weight, and isoelectric point, the biological and molecular function of the identified cellulase protein are given in Table 1. The identified peptides hit the sequence of the master protein at different regions while modification in the carbamidomethyl and their position and amino acid sequence are exemplarily highlighted in Figure 3.

**TABLE 1:** A detailed description and proteome analysis of the cellulase obtained from bacterial isolate (*B. subtilis* CD001) by nano LC-MS/MS using carbamidomethyl modifications.

Accession	Description	Sequence coverage (%)	Peptides	PSMs	Unique peptides	Score sequest HT	MW (kDa)	Calc. pI	Biological process<	Molecular function
AOR98335.1	Cellulase (*B. subtilis*)	46	12	14	12	18.54	55.2	8.47	Metabolic process	Catalytic activity
Annotated sequence			Modifications	Position in protein	PSMs	Missed cleavages	Theo. MH+ [Da]	XCorr: Sequest HT	Amanda score	ChrameRT combined score
[Y].AQIGCGNVTHKFVTLHKPKQGADTYLELGFK.[N]			1xCarbamidomethyl [C5]	[409–439]	1	2	3,457.8049	0.62	9.26	9.26
[K].EAVEAAKELGIYVIIDWHILNDG.[N]				[114–136]	1	1	2,568.33443	2.67	—	—
[K].EMSSLYGNTPNVIYEIAN.[E]				[151–168]	1	0	2014.94263	2.9	23.6	23.6
[N].EPNGDVNWKRDIKPYAEEVISVIR.[K]				[169–192]	1	2	2,827.47372	0.72	—	—
[A].GDGSMNSNQIRPQLQIKNNGNTTVDLK.[D]				[361–387]	1	1	2,942.47486	2	9.98	9.98
[K].GQNVDCDYAQIGCGNVTHKFVT.[L]			2xCarbamidomethyl [C6; C13]	[401–422]	1	1	2,483.10782	3.67	56.59	56.59
[P].KQGADTYLELGFKNGTLGPGASTGNIQLR.[L]				[427–455]	1	2	3,006.56432	3.66	38.51	38.51
[G].NGGVFLDQSREWLKYLDSK.[T]				[266–284]	1	2	2,255.14551	2.8	—	—
[K].QGADTYLELGFKNGTLGPGASTGNI.[Q]				[428–452]	1	1	2,481.22561	2.83	12.98	12.98
[R].SISIFITCLLITLLTMGGMIASPASAAGTKTPVA.[K]			1xCarbamidomethyl [C8]; 2xOxidation [M16; M19]	[4–37]	3	1	3,438.82965	1.14	11.74	11.74
[E].VISVIRKNDPDNIIIVGTGTWSQDVNDAADDQLK.[D]				[187–220]	1	2	3,709.90316	0.87	2.22	2.22
[I].YEIANEPNGDVNWKRDIKPYAEEVISVIR.[K]				[164–192]	1	2	3,417.74374	1.81	1	1

### Characterization of the Partially Purified Cellulase

#### Temperature and pH Effect

**FIGURE 4:**
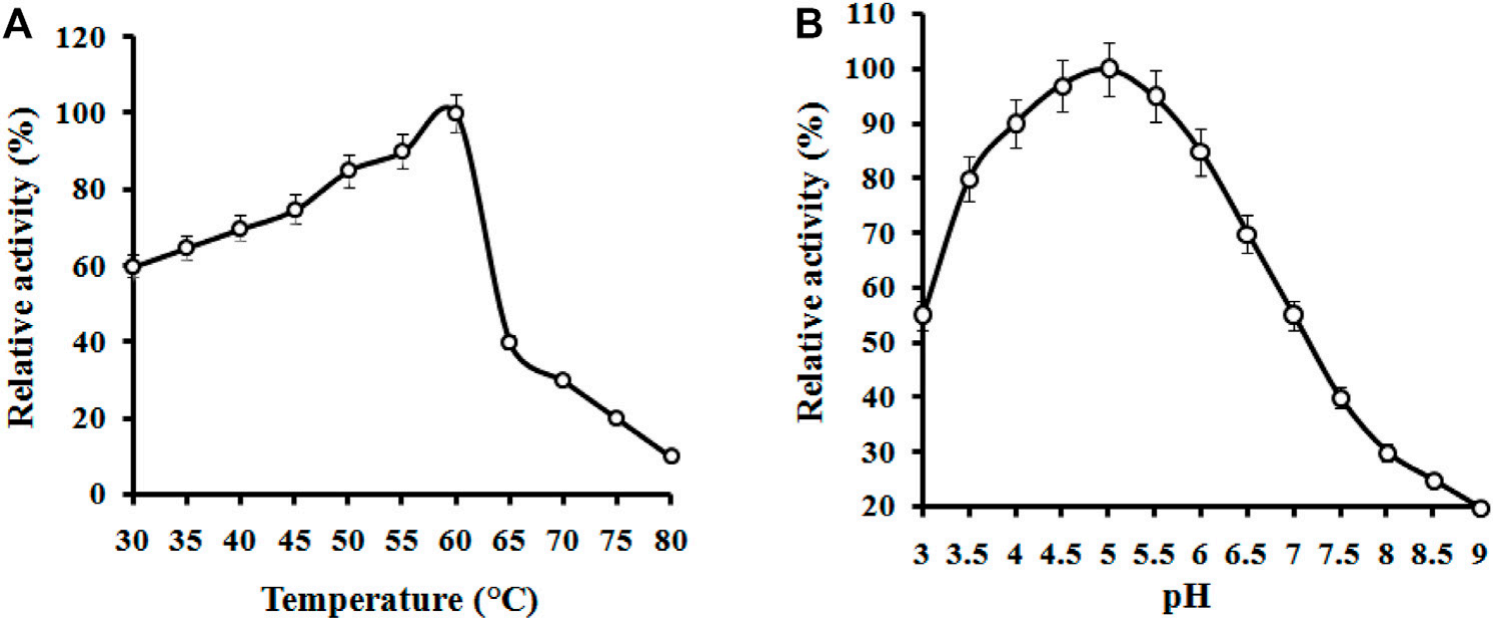
Effects of temperature **(A)** and pH **(B)** on the activity of partially purified *B. subtilis* CD001 cellulase.

The effect of temperature on the CMCase activity was examined at various temperature values ranging from 30°C to 70°C. The activity profile of the enzyme showed its maximum activity between 55 and 60°C (Figure 4). However, the enzyme displayed the highest activity at 60°C, and this temperature was later used for the standard enzymatic assays. A similar value of temperature is required to promote maximal activity for commercial cellulase enzymes. Our findings are in agreement with those of Rawat and Tewari (2012) who have also found 60°C as the most favorable temperature for the enzyme by the bacterium *B. subtilis* LFS3. Vijayaraghavan et al. (2016) also reported the temperature of 60°C as optimum for cellulase activity by a bacterium, *Bacillus halodurans* IND18 isolated from cow dung. The optimum CMCase activity monitored at different temperature levels for various *bacillus* strains was found to be lower than in this study (Abd Elhameed et al., 2020). The optimum temperature (60°C) of the enzyme from *B. subtilis* CD001 was found to be comparably more than other bacterial strains and share similar characteristics when compared to some other strains of bacteria as well. The stability for temperature and pH of previously reported bacterial strains are summarized in Table 2. For diverse industrial applications, relatively high thermostability is an attractive and enviable characteristic of an enzyme. The pH optimum of the cellulase was measured at constant temperature (60°C) and substrate concentration (CMC) of 1.0%. The activity profile of the partially purified enzyme showed its highest activity at pH 5.0, with over 85% of the maximum activity in a pH range of 4.0–5.5; the activity of the enzyme beyond this range was low. Between pH 3.0 to pH 7.0 the activity was confirmed to 50% only. As per the results displayed in Figure 4, in acidic pH 4.0–5.5 conditions, the enzyme showed good stability, whereas its stability in highly alkaline (pH 8.0–10.0) conditions was limited. These results are consistent with other cellulolytic *Bacillus* spp., which showed the optimal pH for cellulase activity towards acidic conditions (Rawat and Tewari, 2012; Li et al., 2020).

**TABLE 2:** Comparison of the properties of cellulase from *B. subtilis* CD001 with those of cellulases reported from other bacterial strains.

Species	Optimum activity		Thermal stability	References
	Temp. (°C)	pH		
*B. subtilis subsp. subtilis* JBS250	60	7	50% of residual activity after 23.5 h incubation at 60°C	Kumar and Singh (2021)
*B. flexus* NT	45	10	28% of residual activity after 30 min incubation at 75°C	Trivedi et al. (2011)
*B. subtilis strain* LFS3	60	4	61% of residual activity after1 h incubation from 40 to 60°C	Rawat and Tewari (2012)
*Cellulosimicrobium cellulans* CKMX1	55	8	50% of residual activity after 30 min incubation at 50°C	Panda et al. (2020)
*Cellulomonas uda* NCIM 2353	50	7	80% of residual activity after 3 h incubation at 70°C	Agrawal et al. (2018)
*B. subtilis Strain* CBS31	50	7.5	45.6% of residual activity after 24 h incubation at 70°C	Sudip et al. (2020)
*B. halodurans* IND18	60	7	100% of residual activity after 1 h incubation at 50°C	Vijayaraghavan et al. (2016)
*B. subtilis* BC1	60	8	74% of residual activity after 1 h incubation at 80°C	Dehghanikhah et al. (2020)
*B.subtilis* YJ1	60	6	90% of residual activity after 30 min incubation at 55°C	Yin et al. (2010)
*B.subtilis* CD001	60	5	80% of residual activity after 72 h incubation at 60°C	(This work)

**FIGURE 5:**
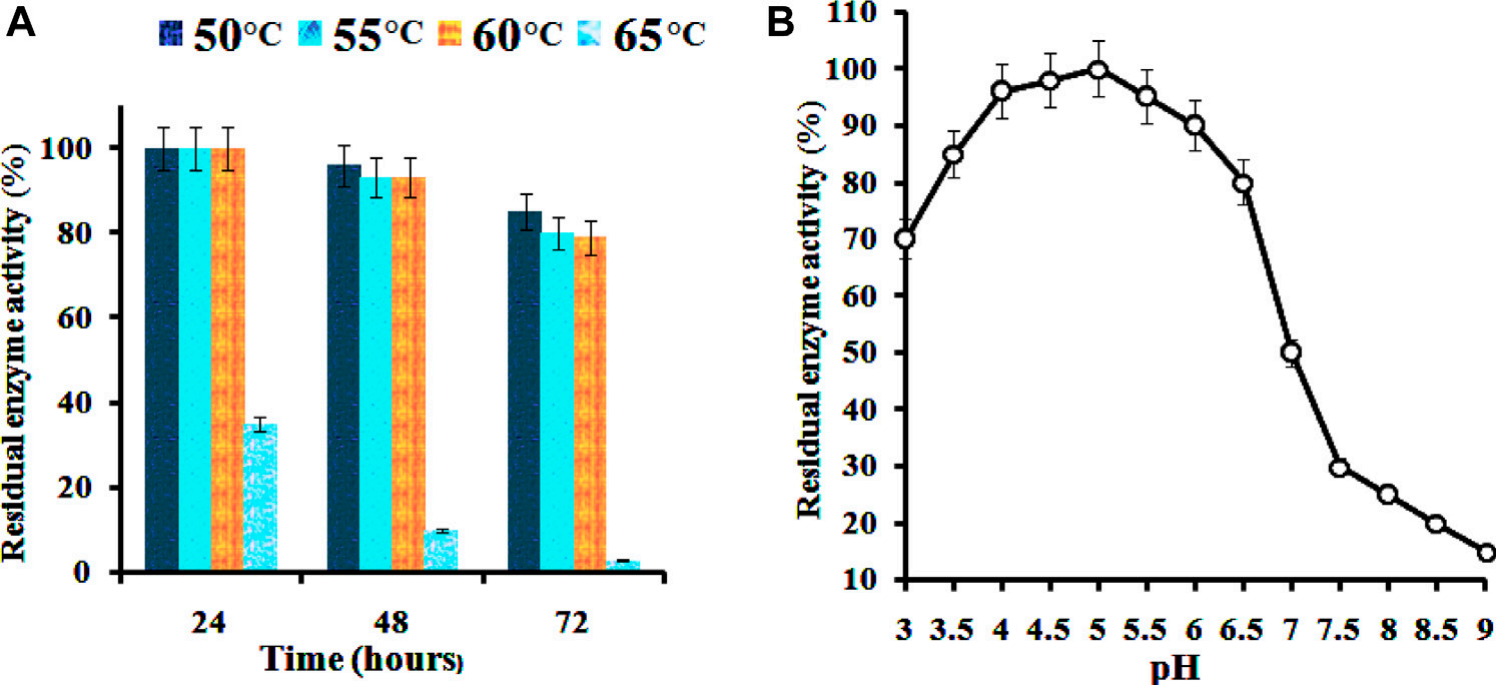
Thermal **(A)** and pH **(B)** stability of the partially purified *B. subtilis* CD001 cellulase.

The enzyme was thermally stable between 50 and 60°C, retaining full activity for up to 24 h and over 75% activity for 72 h (Figure 5). However, the stability of the enzyme declined to 16 and 20% when the incubation time was increased to 48 and 72 h, respectively. At above 60°C, the activity abruptly decreased within 24 h of incubation. The enzyme was found to be stable over a pH range between 4.0–5.5, with 70 and 60% of residual activities, respectively. A hasty decline to 20% was observed at pH 9.0. These results demonstrate the characteristic of the enzyme towards acidic conditions. The enzyme can retain 80% of its maximum activity at pH levels ranging from 3.0 to 6.0, as shown in Figure 5. Many *Bacillus* endoglucanases harbor broader pH stability characteristics. The enzymes operated from previously reported cellulolytic *Bacillu*s spp. have been found to show wider pH stability, as mentioned in Table 2. However, as of now, only a few cellulases from thermoacidophile *Bacillus* species are reported. The popularity gained by the thermostable enzymes in the industrial field is due to their resistance against thermal inactivation (Rawat and Tewari, 2012). Based on the results obtained, the enzyme from *B. subtilis* CD001 may be a potential candidate for industrial lignocellulosic bioethanol production and might contribute to the economy of the production process.

### Kinetic Analysis

**FIGURE 6:**
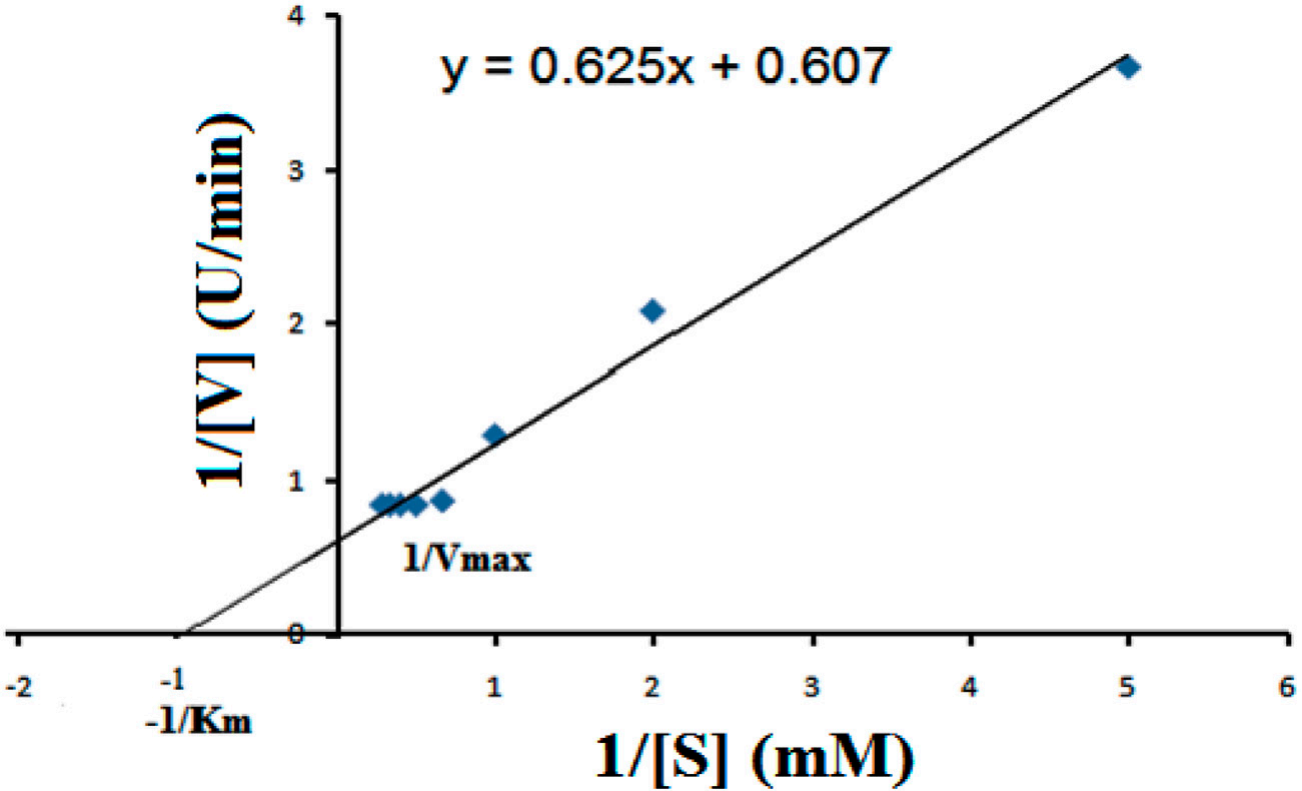
Lineweaver–Burk plot for the determination of Vmax and Km values of the partially purified cellulase from *B. subtilus,* at optimum conditions (60°C, pH 5.0), in the presence of different concentrations of CMC as the substrate.

The kinetic parameters of the enzyme were determined by using CMC as a substrate (0.25–3.0%). Lineweaver–Burk double reciprocal plot was used for the analysis of Vmax and Km values. Initially, the rate of reaction rapidly increased with the concentration of CMC. The enzyme achieved the saturation point at the CMC concentration of 2% (w/v), beyond which the reaction rate stabilized. A closer look at Figure 6 reveals the enzyme’s kinetic properties with Km and Vmax values of 0.996 mM and 1.647 U/ml, respectively. An enzyme with lower Km values signifies their high substrate affinity, i.e., the enzyme can achieve half the maximum rate (Vmax) at a small amount of substrate concentration. The kinetics parameters suggested that the affinity of *B. subtilis* CD001 cellulase to the substrate is higher than that of *B. subtilis* strain CBS31 and *B. subtilis* ASH (Sanghi et al., 2010; Sudip et al., 2020).

### Effect of Metal Ions and Other Chemicals

In most cases, cellulase from the *Bacillus* species exhibited different inhibition and activation with different additives, depending on which cations were used (Trivedi et al., 2011). In this study, various metal ions and other compounds were used to investigate their effect on the partially purified cellulase by performing the assay with different additives. The effect of all the modulators on cellulase activity is given in Table 3. Among ten metal ions tested, the presence of ZnSO_4_ strongly accelerated the reaction medium followed by MnSO_4_ and MgSO_4_^.^ The other ions tested showed an appreciably positive effect on cellulase activity. However, the presence of HgCl_2_ and AgNO_3_ showed a greater decline in the activation process. In response to metal ion binding to amine or carboxylic acid group of amino acids, the metals ions either accelerate or inhibit enzymatic activity. Besides ionic charges, ionic radius size also greatly influences the stability and activity of enzymes (Coolbear et al., 1992; Zeng et al., 2014). The effect of metal ions on the activity of cellulase is not properly elucidated, however, this may occur via redox effects on amino acids that increase or decrease its activity. Also, the effect of metal ions on the activity of enzymes seems to vary between different microorganisms (de Cassia Pereira et al., 2017). In this study, ZnSO_4_ was found to accelerate the activity of the cellulase which correlates well with other studies that demonstrated the positive effect of ZnSO_4_ on cellulase activity (Panthi et al., 2016; Sudip et al., 2020; Kumar and Singh, 2021). The stimulatory effect of Mg^2+^ for the cellulase enzyme was also reported by Sudip et al. (2020) for bacterial cellulase from *B. subtilis* Strain CBS31. However, the inhibitory effect of Hg^2+^ on the cellulase reaction has been mentioned by several other studies (Lee et al., 2008; Rawat and Tewari, 2012). The effect of other additives on cellulase activity is in conformity with the results of Walia et al. (2014). Among the inhibitors, EDTA showed a greater inhibition effect on cellulase activity. While the enzyme was moderately affected by βME, and urea. The results obtained are in accordance with the results obtained by Banerjee et al. (2020). The EDTA acts as a strong inhibitor for cellulase activity because it performs as a chelator metal ion cofactor and inactivates the enzyme (Walia et al., 2014). Among the surfactants tested against cellulase activity, SDS was found to strongly inhibit the enzyme activity. At the same time, Triton X-100 has a minor inhibitory effect on cellulase activity. It has been found that ionic detergents like SDS distort the active site of the protein by non-specific interactions, causing inhibition and conformational changes which result in protein unfolding and instability (Singh, 2017). The detergent Triton X-100 does not react strongly with protein surfaces because it is considered a mild detergent. The same effect of Triton X-100 and SDS on enzyme activity was also reported by Kumar and Singh. (2021).

**TABLE 3:** Effect of modulators on the activity of *B. subtilis* CD001 cellulase.

Chemicals	Relative cellulase activity (%)	Chemicals	Relative cellulase activity (%)
Metal ions (10 mM)		Inhibitors (5 mM)	
None	100	None	100
NaCl	97	β-mercaptoethanol (βME)	71.5
KCl	96	EDTA	21
ZnSO_4_	110	Urea	60
MnSO_4_	101.2	Surfactants (1%)	
MgSO_4_	100	None	100
HgCl_2_	5.6	Triton X-100	85
CaCl_2_	99	SDS	60
FeCl_3_	88		
AlSO_4_	70		
AgNO_3_	9.2		

### Biophysical Characterization of Untreated and Enzyme Pretreated Biomass Residues Using SEM, FTIR, and XRD Analysis

The hydrolytic activity of the crude enzyme with different biomass residues was analyzed by measuring the amount of glucose released using the DNS method. However, the structural and functional changes that occurred on biomass materials via crude enzyme treatment were confirmed by SEM, FTIR, and XRD analysis. The Crude enzyme exhibited significantly higher hydrolytic activity towards CMC, a synthetic soluble cellulosic substrate used as control, followed by SCB, WS, and FP. The percent of sugar molecules released are quantified by a standard glucose curve (Wood et al., 2012) as shown in Figure 1. At the same time, the percentage effect of saccharification that occurred in SCB, WS, and FP was measured after 72 h of the incubation period. The results revealed the maximum saccharification in SCB (40.32%), followed by WS (35.12%), and FP (31.55%). Comparison of the results obtained with previous studies shows that earlier also the action of bacteria and their enzyme on biomass residues have resulted in an increased percent saccharification rate. Tsegaye et al. (2018) reported a 28.49% saccharification rate of wheat straw after biotreatment with bacterial strain *Ochrobactrum oryzae* BMP03. About 54.38% biomass saccharification rate was observed after cellulase treatment from bacteria, *B. subtilis* K-18 (Irfan et al., 2017). An individual and combined application of different bacterial strains resulted in an efficient saccharification rate in SCB (Thite and Nerurkar, 2020).

### SEM

**FIGURE 7:**
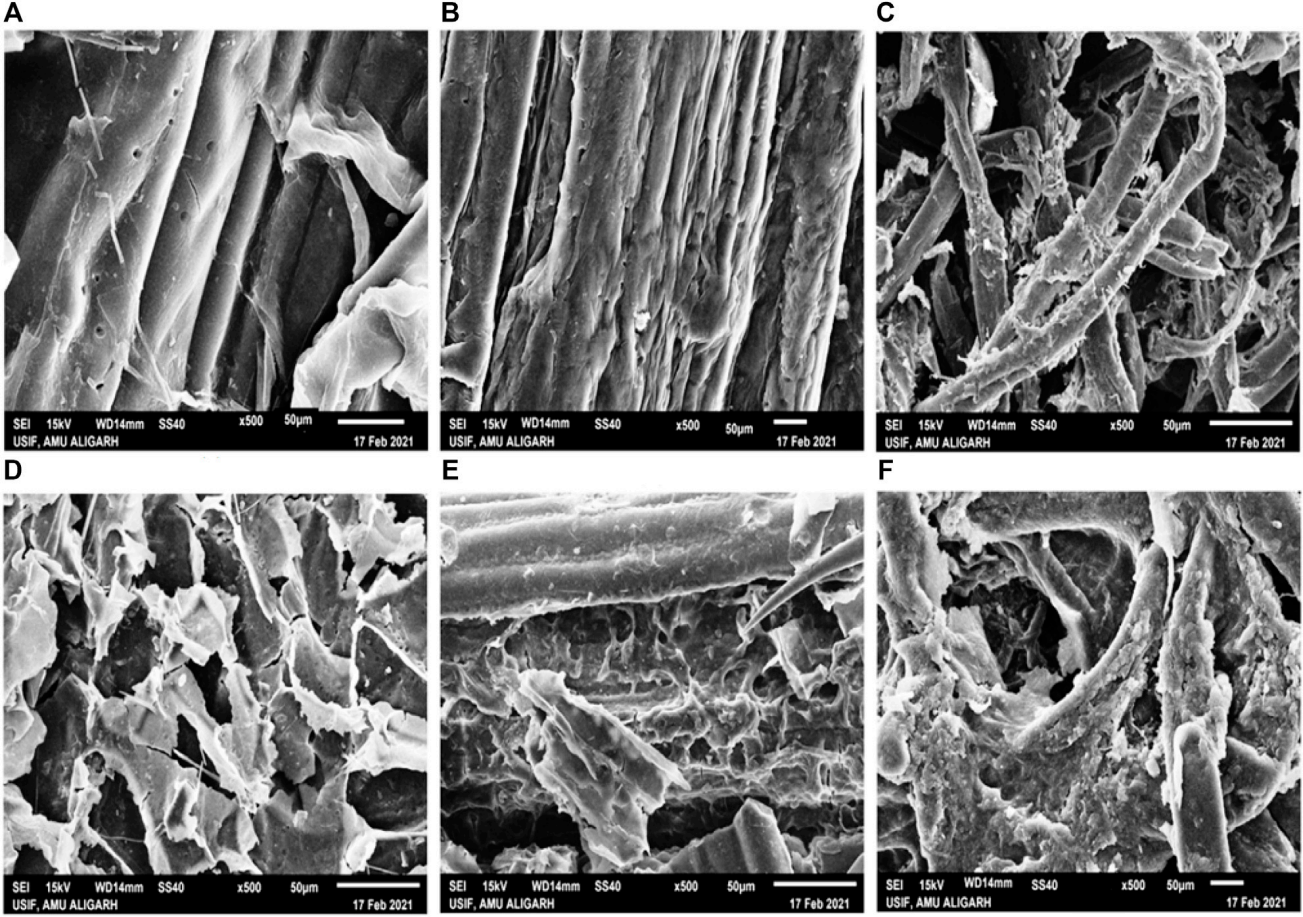
Scanning electron microscopy (SEM) of untreated biomass, SCB, WS, and FP **(A–C)**, and biomass residues, SCB, WS, and FP **(D–F)** treated with *B. subtilis* CD001 cellulase respectively.

Scanning electron microscopy was carried out to study the morphological changes of SCB, WS, and FP caused by crude cellulase treatments. Morphological modifications of enzyme-treated SCB, WS, and, FP were observed in comparison with untreated SCB, WS, and, FP. In the case of raw untreated SCB, WS, and, FP the micrographs showed parallel strips of vascular bundles surrounded by fragile pith cells with flaky surfaces (Figures 7A–C). The organized and rigid structure with a smooth surface observed in case of raw biomass indicated no change in the hydrolytic process. After crude enzyme treatment, the distortion of the surface along with the separation of fibrous vascular bundles was seen in all three cases (Figures 7D–F). This disruption of the fibers confirms that the enzymatic treatment resulted in extensive damage and formed disorganized fibers with large pore size. The structural analysis of biomass residues suggested that the crude enzyme treatment caused structural changes in biomass residues which may be referred to as its lignin and/or hemicellulosic components alterations (Malik et al., 2021). In the case of SCB, the treatment has altered the architecture of the cell wall matrix components or removed the cellulose, lignin, and hemicellulosic components. Following bacterial and its enzyme treatment, similar alterations of several cracks were seen in SCB with a large number of holes, disordered fibers, and broken parallel stripes (Namnuch et al., 2021; Prajapati et al., 2020). A resemblant phenomenon was observed in other studies conducted on SCB hydrolysis with crude enzyme treatment (Matei et al., 2020; Ramarajan and Manohar, 2017). The biodegradability of WS was also analyzed by SEM before and after crude cellulase treatment. A study by Tsegaye et al. (2018) observed a compact, rough, and ordered structure in raw WS while a disorganized and loosened matrix of WS was noted after the treatment. Similar morphological changes on WS were observed by Pedersen et al. (2020) on enzymatic treatment. Zheng et al. (2018) found that the enzymatic treatment completely decimated WS after acid- and alkali-pretreatments which then confirmed that most of the cellulose was degraded by the enzyme. These results also correlate with the study by Irfan et al. (2016) which indicates the degradation of lignin and hemicelluloses content after treatment with *Saccharomyces cerevisiae*. However, in this study same structural alterations were seen in WS without any specific pretreatment method applied. All the changes that occurred were attributed to the application of crude enzyme only. Figures 7C,F shows the structural changes that occurred in FP before and after crude enzyme treatment. Though the lesser saccharification percentage was noticed in FP than SCB and WS; however, a clear structural change can be seen in FP after the application of enzyme treatment. This data agreed with the observations from SEM analysis on cellulase treatment to FP by Beyene et al. (2018). Hence these findings demonstrate that the direct application of crude cellulase without any extraneous pretreatment could destabilize the network of cellulose, hemicellulose, and lignin, therefore removing some of the external fibers of biomass residues and accelerating the biodegradation process (Du et al., 2016).

### FTIR

**FIGURE 8:**
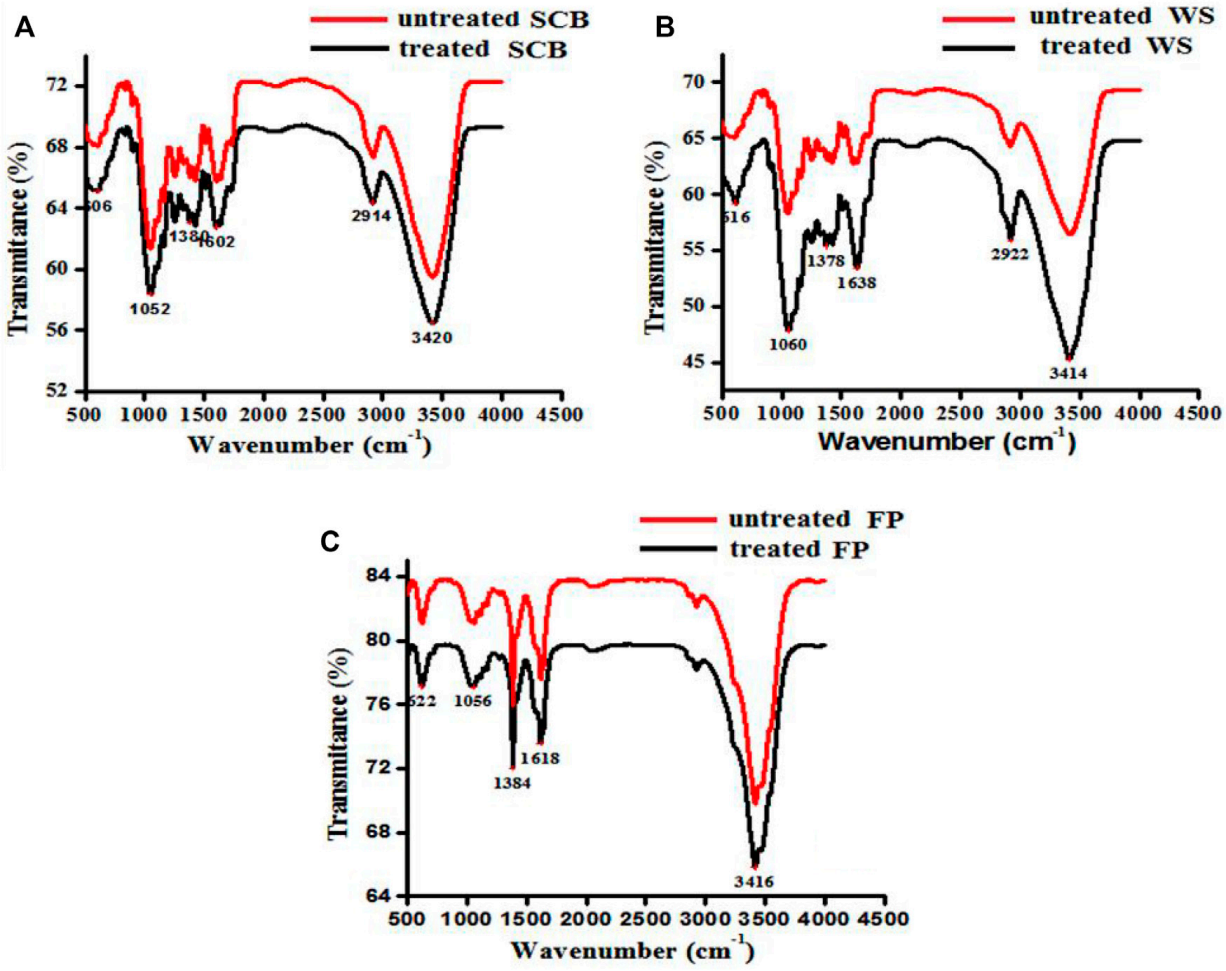
FTIR spectra of untreated SCB, WS, and FP [**(A–C)** denoted in red] and cellulase treated [**(A–C)** denoted in black] biomass materials, respectively.

FTIR analysis was carried out to examine the chemical changes that occurred in SCB, WS, and FP before and after crude enzyme treatment. Figure 8 illustrates the FTIR profiles of SCB, WS, and FP. The broad absorption band between 3,420 and 3,414 cm^−1^, which exhibited the greatest intensity at approximately 3,420 cm−^1^, is related to O-H stretching vibration (Baramee et al., 2020). The peak at 2,922–2,912 cm−^1^ depicting the CH stretching found in all crude enzyme-treated biomass residues corresponds to cellulose/hemicellulose stretching (da Silva et al., 2018). The absorption bands obtained around 1,052, 1,060, and 1,056 correspond to the β-1,4-glycosidic linkage, and these results showed close proximity with the results obtained by Baramee et al. (2020) and Li et al. (2014). A small peak around 1,600–1,640 cm^−1^ is related to the C=O around the aromatic ring and was found less intense in enzyme-treated than untreated samples (Saini et al., 2020). In literature, similar changes at these wavenumbers have been reported after the enzyme and chemical-mediated delignification process (Kuhad et al., 2010; Rajak and Banerjee, 2015; Thite and Nerurkar, 2019). Bands observed near 1,380 cm^−1,^ and 1,052 cm^−1^ appear to be characteristic of C-H cellulose and hemicelluloses (Rajak and Banerjee, 2015). The peaks around 1,616, 1,638, 1,602, 1,378, 1,384, 1,380, 1,050, and 1,080 cm^−1^ attributed to chemical bonds and structures present in biomass components that were significantly affected by enzyme treatment when compared with untreated raw biomass. The difference in the peaks of untreated and pretreated SCB, WS, and FP showed variations in their chemical and structural properties. Shift or disappearance occurred in the regions of SCB, WS, and FP by the application of crude enzyme treatment is indicative of their degradation by *B. subtilis* CD001 cellulase. A reduction of the contents of various functional groups in cellulose and hemicelluloses and the disappearance of some bonds indicate that these materials are being deconstructed intramolecularly.

### XRD

**FIGURE 9:**
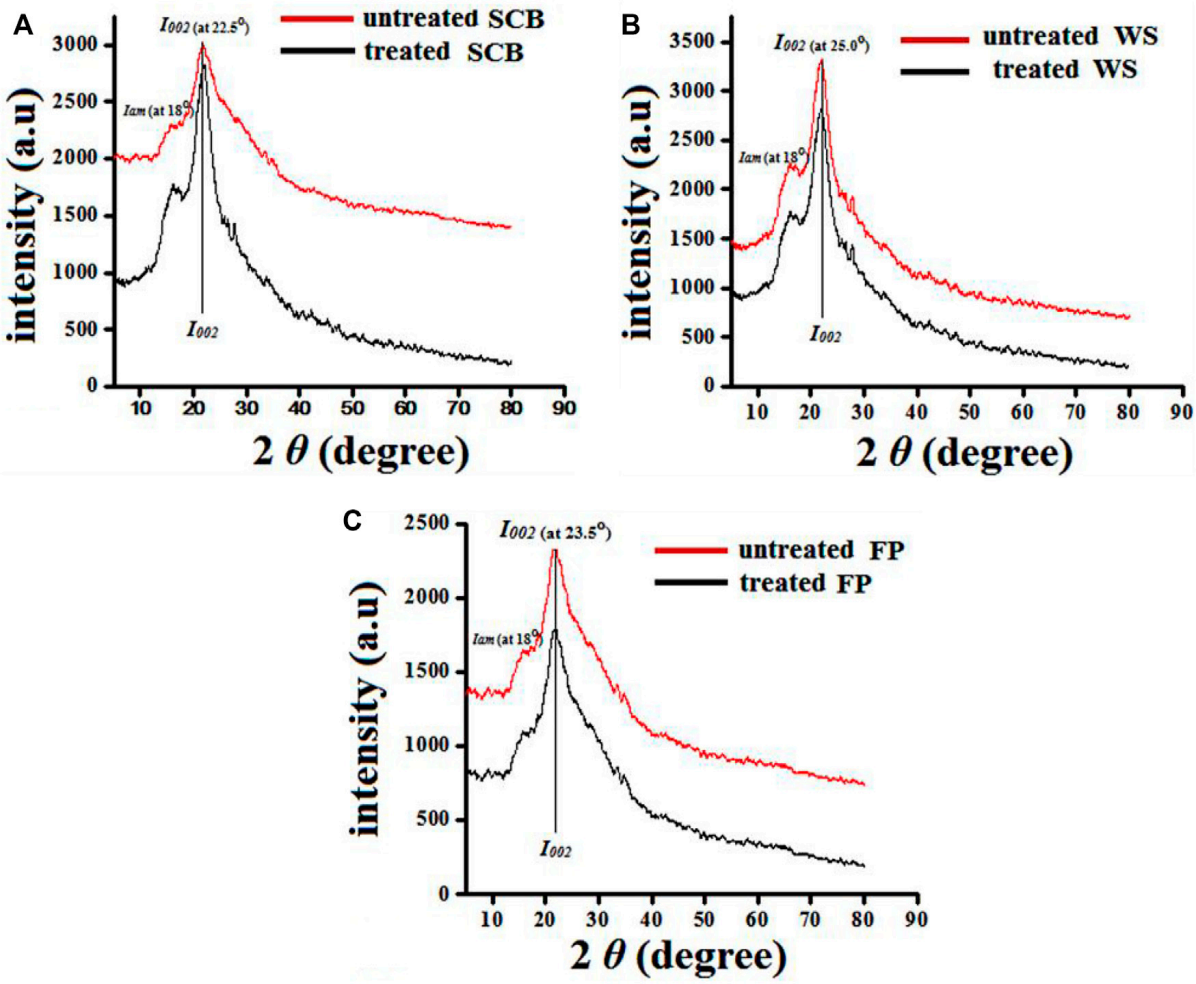
X-ray diffraction analysis of untreated, SCB, WS, and FP [**(A–C)** denoted in red] and enzyme-treated, SCB, WS, and FP [**(A–C)** denoted in black] respectively.

Cellulose crystallinity is an important factor to evaluate the effect of saccharification in terms of increased % crystallinity brought by microbes and their enzymes. Biodegradability of the biomass after valorization depends on the cellulose crystallinity together with enzymes (Rajak and Banerjee, 2015). Thus, an XRD analysis was performed to understand the effect of crude cellulase treatment on the crystallinity of SCB, WS, and FP. Figure 9 shows the XRD patterns of SCB, WS, and FP before and after crude cellulase treatment. After the treatment, amorphous regions in biomass residue salinities were hydrolyzed, causing the peaks to gradually improve. Compared to the CrI of raw biomass (SCB 55.21%, WS 47.11%, and FP 47.20%) the CrI of crude enzyme-treated biomass was considerably higher for SCB 60.12%, WS 49.5%, and FP 49.34%, respectively. These results describe that the hemicellulose components are possibly depolymerized by removing non-cellulosic polysaccharides and disrupting the intra-and interchain hydrogen bonds of cellulose fibrils (Chutani and Sharma, 2015; Trevorah et al., 2021). Similar observations have been reported on WS hydrolysis after enzymatic exposure by Zheng et al. (2018). Prajapati et al. (2020) reported that compared to raw SCB (58.41%), the CrI of the SCB sample increased to 63.91% after the enzymatic treatment from *Aspergillus tubingensis* NKBP-55. The increase in CrI due to preferential degradation of amorphous regions in SCB is also reported by Ghosh et al. (2019). In light of this, the enzyme treatment could result in partial degradation of cellulosic components, which increased the crystallinity of the biomass and cellulose conversation rate. The characteristic peaks at 2θ = 22.5–25° and around 18° are related to the crystalline region (I_00_2) and amorphous region (I_am_), respectively. However, the net increase in CrI of the enzyme-treated SCB, WS, and FP is due to the hydrolysis of amorphous regions of cellulose and hemicellulose (Maeda et al., 2011; Zheng et al., 2018). A report by Chen et al. (2007) shows that after treatment with cellulase (Cel6A), bacterial cellulose has an increase in CrI of 2.6%. Similarly, Wang et al. (2006) observed an increase in CrI of 2% in cotton after treatment with cellulase for 6 days. In the present study, the increment of 4.91, 2.39, and 2.14% for SCB, WS, and FP indicated the high efficacy of *B. subtilis* cellulase towards amorphous biomass residues.

## Conclusion

A potential cellulose degrading enzyme from *B. subtilis* CD001 was characterized and studied for its possible hydrolyzing capability for disintegrating the cellulosic biomass residues. The crude enzyme exhibited the properties of CMCase, FPase, and amylase that confirmed its broader substrate specificities, important for biomass saccharification. Characterization studies of the enzyme revealed it as an acidothermophilic cellulase retaining full activity up to 24 h at 60°C and keeping 80% of its maximum activity at a pH range of 3.0–6.0. Enzymatic treatment of SCB, WS, and FP using crude enzyme extract rich in cellulase produced from *B. subtilis* CD001 demonstrated its ability to disintegrate and hydrolyze biomass structures. It is evident from the structural analysis of enzyme saccharified biomass residues that the cellulase from this strain of *B. subtilis* is an efficient saccharifying agent signifying its ability for structural breakdown and hydrolysis. Therefore, the isolate is expected to be economically beneficial for enhanced enzymatic hydrolysis of biomass residues for possible bioethanol production.

## Data Availability

The original contributions presented in the study are included in the article/Supplementary Material, further inquiries can be directed to the corresponding author.
